# Evidence for disulfide bonds in SR Protein Kinase 1 (SRPK1) that are required for activity and nuclear localization

**DOI:** 10.1371/journal.pone.0171328

**Published:** 2017-02-06

**Authors:** Maria Koutroumani, Georgios E. Papadopoulos, Metaxia Vlassi, Eleni Nikolakaki, Thomas Giannakouros

**Affiliations:** 1 Laboratory of Biochemistry, Department of Chemistry, Aristotle University, Thessaloniki, Greece; 2 Department of Biochemistry and Biotechnology, University of Thessaly, Mezourlo, Larisa, Greece; 3 Institute of Biosciences & Applications, National Centre for Scientific Research "Demokritos", Athens, Greece; University of Toronto, CANADA

## Abstract

Serine/arginine protein kinases (SRPKs) phosphorylate Arg/Ser dipeptide-containing proteins that play crucial roles in a broad spectrum of basic cellular processes. The existence of a large internal spacer sequence that separates the bipartite kinase catalytic core is a unique structural feature of SRPKs. Previous structural studies on a catalytically active fragment of SRPK1, which lacks the main part of the spacer domain, revealed that SRPK1 remains in an active state without any post-translational modifications or specific intra-protein interactions, while the spacer domain is depicted as a loop structure, outside the kinase core. Using systematic mutagenesis we now provide evidence that replacement of any individual cysteine residue in the spacer, apart from Cys414, or in its proximal flaking ends of the two kinase catalytic domains has an impact on kinase activity. Furthermore, the cysteine residues are critical for nuclear translocation of SRPK1 in response to genotoxic stress and SRPK1-dependent splicing of a reporter gene. While replacement of Cys207, Cys502 and Cys539 of the catalytic domains is predicted to distort the kinase active structure, our findings suggest that Cys356, Cys386, Cys427 and Cys455 of the spacer domain and Cys188 of the first catalytic domain are engaged in disulfide bridging. We propose that such a network of intramolecular disulfide bonds mediates the bending of the spacer region thus allowing the proximal positioning of the two catalytic subunits which is a prerequisite for SRPK1 activity.

## Introduction

SRPKs constitute a subfamily of serine-threonine kinases that specifically phosphorylate serine residues residing in arginine-serine dipeptide motifs, known as RS domains [1–3]. The mammalian genome encodes for more than 100 RS domain-containing proteins, almost half of which are implicated in regulating mRNA processing and the remaining half in a variety of other critical cellular activities [1, 4]. These fundamentally important functions render SRPKs essential for viability of proliferating cells.

**Fig 1:**
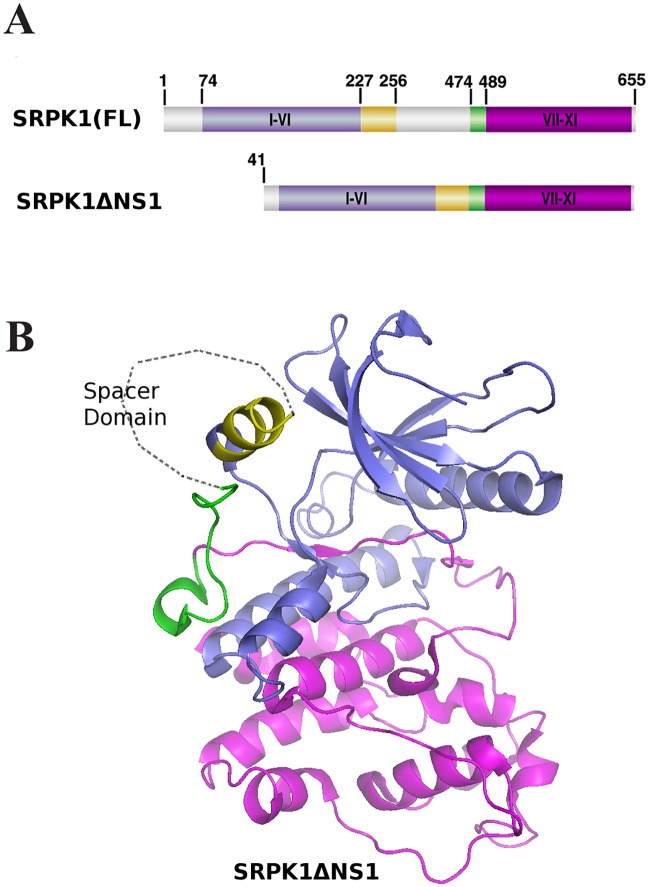
Structural features of SRPK1. (A) Domain organization of SRPK1 as presented in ref. 8. The N- and C-terminal conserved kinase subdomains are colored purple and magenta, respectively. The N- and C-terminal spacer regions are colored yellow and green, respectively, while the remaining spacer domain is colored gray. SRPK1(FL): full-length SRPK1; SRPK1 ΔNS1: the crystallized fragment of SRPK1. (B) Overall structure of SRPK1 ΔNS1 as presented in refs 8 and 16. The coloring of the domains is as in (A). Note that the spacer domain is depicted as a loop outside the kinase core.

Most of our knowledge regarding SRPKs comes from studies on the mammalian SRPK, SRPK1. SRPK1 is required for embryonic development in the mouse since knocking it out resulted in a lethal phenotype [5]. SRPK1 contains two well-conserved kinase domains that are separated by a long divergent spacer sequence, a feature common among tyrosine kinases, but rare in serine/threonine kinases ([1–3], see Fig 1A). The spacer domain (aa 227–489), which is almost as large as the sum of the two catalytic domains (aa 74–227 the first; aa 489–665 the second), is practically needless for kinase activity [6–8]. In addition to the spacer domain, SRPK1 contains an N-terminal extension (aa 1–74), which is not conserved among SRPKs and the deletion of which does not also seem to affect the catalytic activity of the kinase [8]. Due to the rather auxiliary role of the spacer and the N-terminal domain in SRPK1 activity, and to facilitate crystallization, these two domains were omitted in the currently available crystal structure ([8], see Fig 1B). According to this structure, SRPK1 adopts a bi-lobal conformation found in most eukaryotic protein kinases [8]. The N-terminal small lobe is composed mostly of β-strands and contains the ATP binding pocket, while the C-terminal larger lobe is composed mostly of α- helices and contains the substrate binding site. Only two small helices (aS1, aa 227–256 and aS2, aa 474–489) from the spacer domain were included in the reported crystal structure which were shown to interact with the small and the large lobe of the kinase, respectively. Despite the fact that biochemical and molecular dynamics data demonstrated that the above interactions were important for the global stabilization of SRPK1 they were not required for activity [8].

While many protein kinases require specific post-translational modifications or additional interactions with scaffolding or adaptor proteins for optimal activity, SRPK1 is regarded as a constitutively active kinase. Structural studies revealed that the activation loop of SRPK1, which is rather short and lacks a regulatory phosphorylation site, adopts a stable conformation that permits ready access of substrates to the active site [2]. This segment seems to be resilient to inactivation, since mutations of a number of residues are well-tolerated due to new contacts mediated by alternative residues [2, 8]. Furthermore, the bi-lobal structure of the kinase facilitates several short-range interactions between the large and small lobes, thus maintaining the structural integrity and functionality of the activation loop [2]. A key experimental finding strengthening these structural observations is that SRPK1, when expressed in bacteria which lack the post-translational modification machinery of eukaryotic cells, is active and efficiently phosphorylates its substrates [9, 10]. However, there are also reports showing that the non-catalytic regions of SRPK1, which were omitted in the crystallization process, may affect phosphorylation of its substrates. More specifically, the N-terminus of SRPK1 may enhance catalysis either through phosphorylation by CK2 [11] or by increasing the rate-limiting ADP release step in the kinase reaction, in cooperation with a small helical segment of the spacer (aS1), proximal to the first catalytic domain [12]. Conversely, binding of the nuclear scaffold proteins SAFB1 and SAFB2 presumably to the N-terminus of SRPK1 was shown to repress activity [13].

Even though the spacer seems to have a rather insignificant role in SRPK1-mediated catalysis, this domain is the critical modulator of SRPK1 subcellular localization, as deletion of this sequence changes the distribution pattern of the kinase from mainly cytoplasmic to exclusively nuclear [7, 14]. The nuclear accumulation of SRPK1 causes the aggregation of splicing factors and possibly affects gene expression [7], while nuclear translocation of Sky1 in *S*. *cerevisiae* was shown to provoke inhibition of cell growth [14]. The cytoplasmic sequestration of SRPK1 is mediated through interactions of the spacer region with specific members of the molecular chaperone family [7, 15]. Unlike the kinase core, the large spacer domain was predicted to lack regular secondary structure and most likely to be unfolded, thus providing an intrinsically disordered platform for chaperone interactions [16]. The association of SRPK1 with molecular chaperones is modulated by upstream signal(s) resulting in the release and subsequent translocation of the kinase to the nucleus. The major signal transduction pathway reported, to date, for regulated nuclear translocation of SRPK1 is the EGF-Akt-SRPK1 pathway [17]. Activated Akt, in response to EGF signaling, binds to and induces SRPK1 autophosphorylation that modifies the SRPK1-chaperone complexes, leading to enhanced nuclear translocation of the kinase and alterations to the splicing program.

In all simplified models of SRPK1 the spacer region is depicted as a loop, outside the kinase core ([8, 16], see Fig 1B). Although the spacer sequence is currently considered as a disordered segment it remains rather unclear whether it may adopt any regular folded structure. Here we initially noticed that the absence of dithiothreitol (DTT) resulted in a shift of the SRPK1 band towards lower molecular weights, upon SDS gel electrophoresis, whereas no such shift was obtained when an active fragment of SRPK1 lacking the spacer sequence was analyzed. Furthermore, treatment with reducing agents had a profound inhibitory effect on kinase activity. Mutational analysis strongly suggests the existence of multiple disulfide bonds in the active form of SRPK1, involving Cys356, Cys386, Cys427 and Cys455 of the spacer and most probably Cys188 of the first catalytic domain.

## Materials and methods

### Plasmid construction and expression of recombinant proteins

The pGEX-2T and pFLAG—CMV-2 vectors expressing wild type SRPK1 have been described previously [10, 18]. *SRPK1Δspacer* encoding a deletion mutant of SRPK1 which lacks the spacer domain (aa 256–475) was amplified by PCR from plasmid pGEX-2T-SRPK1Δspacer [13] with the upstream primer 5’-GCCGGAATTCAATGGAGCGGAAAGTGCTTGCG-3’ and downstream primer 5’-CCCGGGATCCGGAGTTAAGCCAAGGGTGCCG-3’ and ligated into the EcoRI/ BamHI site of pFLAG—CMV-2. Point mutations were inserted in SRPK1 by site-directed mutagenesis using the QuickChange^®^ Lightning kit (Stratagene) following the manufacturer's instructions. The following primers (s, sense; a, antisense) were used to mutate the respective cysteine residues to alanine (Cys188 and Cys539) or glycine (Cys207, Cys356, Cys386, Cys414, Cys427, Cys455 and Cys502):

Cys188 (s): 5’-CAGGGGCTTCCACTGCCTGCTGTCAAAAAAATTATTC-3’

Cys188 (a): 5’-GAATAATTTTTTTGACAGCAGGCAGTGGAAGCCCCTG-3’

Cys207 (s): 5'-GATTATTTGCATACCAAGGGCCGTATCATCCACACTG-3'

Cys207 (a): 5'-CAGTGTGGATGATACGGCCCTTGGTATGCAAATAATC-3'

Cys356 (s): 5'-GGTGCAGCAGAAATTAATGGCAATGGAGTGATTGAAG-3'

Cys356 (a): 5'-CTTCAATCACTCCATTGCCATTAATTTCTGCTGCACC-3'

Cys386 (s): 5'-GGATCTACATAATGCTAATGACGGTGATGTCCAAAATTTGAATC -3'

Cys386 (a): 5'-GATTCAAATTTTGGACATCACCGTCATTAGCATTATGTAGAT CC -3'

Cys414 (s): 5'-TCTCAAGAAACAGACTCTGGTACACCTATAACATCTGAG-3'

Cys414 (a): 5'-CTCAGATGTTATAGGTGTACCAGAGTCTGTTTCTTGAGA-3'

Cys427 (s): 5'-GTGTCAGACACCATGGTGGGCCAGTCTTCCTCAACTG-3'

Cys427 (a): 5'-CAGTTGAGGAAGACTGGCCCACCATGGTGTCTGACAC-3'

Cys455 (s): 5'-ATTCGGGCAGAGATACCCGGTGAAGATGAACAAGAGC-3'

Cys455 (a): 5'-GCTCTTGTTCATCTTCACCGGGTATCTCTGCCCGAAT-3'

Cys502 (s): 5'-GCTGACCTTGGAAATGCTGGTTGGGTGCACAAACATTTC-3'

Cys502 (a): 5'-GAAATGTTTGTGCACCCAACCAGCATTTCCAAGGTCAGC-3'

Cys539 (s): 5'-CATTTGGAGCACGGCAGCCATGGCCTTTGAACTG-3'

Cys539 (a): 5'-CAGTTCAAAGGCCATGGCTGCCGTGCTCCAAATG-3'

All the mutated cDNAs were sequenced to rule out unwanted mutations. GST-LBRNt(62–92) which contains the RS region of Lamin B Receptor (LBR) was produced in bacteria and purified using glutathione-Sepharose beads as previously described [19].

### Cell culture and transfection

HeLa and 293T cells were cultured in DMEM medium supplemented with 10% (v/v) fetal bovine serum (FBS) and antibiotics, while K562, MEL, MOLT-4 and JM1 cells were maintained in RPMI medium containing 10% FBS and antibiotics. Cells were incubated at 37°C with 5% CO_2_. Transfections of plasmids expressing wild type SRPK1 and derived mutants were done with the Xfect^™^ transfection kit (Clontech Laboratories, Inc.), according to the manufacturer's instructions and cells were collected after 48 h. For 293T cells, 2x10^5^ cells were plated in 12-well plates and 1.5 μg of plasmid DNA was diluted with Xfect Reaction Buffer to a final volume of 100 μl and then added to 0.5 ml DMEM (without FBS). For HeLa cells, 7x10^4^ cells were plated in 24-well plates and 1 μg of plasmid DNA was diluted with Xfect Reaction Buffer to a final volume of 50 μl and added to 0.25 ml DMEM (without FBS). Following 4 h incubation, nanoparticle complexes were removed from both 293T and HeLa cells by aspiration and 2 ml (12-well plates) or 1 ml (24-well plates) fresh complete growth medium were added. For immunofluorescence and splicing studies, HeLa cells were treated with 50 μg/ml 5-fluorouracil (5-FU) for 36 h and 24 h respectively, prior harvesting.

### Protein detection by SDS-PAGE and Western blotting

Cells were lysed in 200 μl of 1% Triton buffer (1% Triton X-100, 50 mM Tris-HCl, pH 7.5, 150 mM NaCl, and 1 mM PMSF). Whole cell extracts were clarified by centrifugation for 15 min at 13,000 g in a microcentrifuge, and analyzed on 10% SDS-PAGE. For non-reducing conditions, DTT (90 mM) was omitted from the protein loading buffer. Western blotting was performed with an anti-SRPK1 monoclonal antibody (BD Biosciences) or the M5 anti-FLAG monoclonal antibody (Sigma), an alkaline phosphatase-coupled goat anti-mouse secondary antibody, and 5-bromo-4-chloro-3-indolyl phosphate/nitro blue tetrazolium substrate. Gel loading was adjusted to give equivalent cell numbers in each lane. Coomassie Brilliant Blue (CBB) staining of bacterially produced recombinant proteins was performed according to standard procedures.

### In vitro kinase assays

Kinase assays were carried out in a total volume of 25 μl containing GST-SRPK1 or GST-SRPK1 cysteine mutants (0.5 μg each), 2 μg GST-LBRNt(62–92) as substrate, 25 μM ATP, and 1 μCi of [γ-^32^P]ATP for 30 min at 30°C. As kinase source, FLAG-SRPK1 and FLAG-SRPK1 derived mutants, immunopurified from transiently transfected 293T cells were also used. For immunoprecipitation, cells were lysed in 1% Triton buffer, and FLAG-SRPK1 proteins were collected on 20 μl of protein A-Sepharose coupled to 2 μg of M5 anti-FLAG antibody. Immunoprecipitates were washed four times in lysis buffer and twice in kinase reaction buffer (12 mM Hepes pH 7.5, 10 mM MgCl_2_). Phosphoproteins were detected by autoradiography using Super RX (Fuji medical X-ray film), and signals were quantified by excising the radioactive bands from the gel and scintillation counting.

### Immunofluorescence microscopy

HeLa cells transfected with plasmids encoding FLAG-SRPK1 and derived cysteine mutants were grown on glass coverslips and treated with 50 μg/ml 5-FU or DMSO, as control, for 36 h. After the incubation period the cell coverslips were fixed with 4% paraformaldehyde in phosphate-buffered saline (PBS) pH 7.4 for 20 min at room temperature and permeabilized with 0.2% Triton X-100 in PBS for 12 min. We used 0.5% fish skin gelatin in PBS to block nonspecific binding during antibody staining. Probing with the primary (anti-FLAG, diluted 1:1500) and secondary (FITC-conjugated goat anti-mouse, diluted 1:400; Molecular Probes) antibodies and DNA staining (propidium iodide) were performed as previously described [20]. After 3x washing, the coverslips were mounted in 90% glycerol and visualized in a Nikon confocal microscope using the EZ-C1 3.20 software. Background corrected nuclear/cytoplasmic SRPK1 fluorescence ratios were calculated from mean fluorescence intensity in nucleus and cytoplasm using ImageJ software (http://www.bio-protocol.org/e1018). In each case, data represent the means ± SE of measurements from 20–30 cells.

### *In Vivo* splicing of a reporter gene

The pSVIRB vector [21], kindly provided by Athena Andreadis (University of Massachusetts Medical School) was used in our *in vivo* splicing assays. This vector contains rat insulin exons 1, 2 and 3 and the respective introns, flanked by SV40 promoter/enhancer regions and insulin transcription terminators. 3 × 10^4^ HeLa cells were transfected with 1.65 μg of the reporter gene together with increasing amounts (0, 0.25, 0.3, 0.35, 0.5 and 0.7 μg) of plasmid DNA encoding FLAG-SRPK1 and derived cysteine mutants, using the Xfect^™^ transfection kit. Cells were treated with 50 μg/ml 5-FU for 24 h, prior harvesting. RNA was isolated, 48 h following transfection, using the RNeasy mini kit (Qiagen) and one μg of it was reverse transcribed using the M-MLV RT kit (Invitrogen), according to the manufacturer’s instructions. Part of this reaction mixture (1 μl) was diluted to a final volume of 50 μl, the concentrations of the buffer and dNTPs were adjusted for PCR and the mixture was initially denaturated at 94°C for 2 min and then amplified for 30 cycles using the DyNAzyme polymerase (Finnzymes OY). PCR conditions were: denaturation at 94°C for 1 min, annealing at 65°C for 1 min, extension at 72°C for 1 min and a final extension at 72°C for 5 min. The primers used were INS1 (sense): 5’-CAGCTACAGTCGGAAACCATCAGCAAGCAG-3’ and INS3 (antisense): 5’-CACCTCCAGTGCCAAGGTCTGAAGGTCACC-3’. Amplified products were resolved by electrophoresis through 1% agarose gel and ethidium bromide staining. The obtained bands (upper band intron inclusion, size ~ 1 kb; lower band intron exclusion, size ~0.3 kb) were quantified using ImageJ software.

### Structure/function predictions

The known crystal structure of an active fragment of SRPK1, and more precisely the PDB entry with code: 3BEG [22], was used to map the cysteines of the SRPK1 catalytic domain in order to evaluate the structural/functional consequences of their mutation. This particular crystal structure was chosen because it provides structural information for the recognition/binding of its protein substrates [22]. The same crystal structure was used to model the C188A mutant, which was subsequently used as the starting conformation for a molecular dynamics (MD) simulation in explicit water. The solvated system (8110 water molecules) was first energy minimized and then subjected to a 20 ns-long NPT MD simulation at 300K with the Particle Mesh Ewald (PME) algorithm for handling electrostatics. The final SRPK1 188C mutant model (averaged protein conformation over the last 500 ps of the MD trajectory) was subjected to energy minimization. The MD program NAMD [23] and the CHARMM force field for proteins and nucleic acids have been used for the simulations. The final model was structurally aligned on the crystal structure and then examined for possible significant local structural changes. Molecular model illustrations were rendered using PyMOL (The PyMOL Molecular Graphics System, Schrödinger, LLC).

## Results

### Effect of reducing reagents on SRPK1 conformation and activity

**Fig 2:**
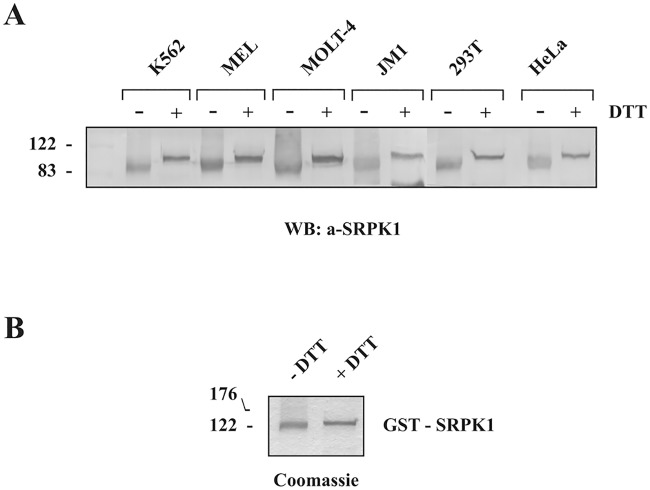
Effect of reducing agents on SRPK1 electrophoretic mobility. (A) Extracts from K562, MEL, MOLT-4, JM1, 293T and HeLa cells were analyzed under non-reducing conditions (i.e., without DTT) or reducing conditions (in the presence of 90 mM DTT) on 10% SDS-polyacrylamide gels. The proteins were then transferred to nitrocellulose, and SRPK1 was detected with an anti-SRPK1 monoclonal antibody. (B) GST-SRPK1 was analyzed in the absence or in the presence of 90 mM DTT on 10% SDS-polyacrylamide gels and stained with Coomassie Blue.

It is well known that redox conditions can be manipulated to alter protein structure and function in vitro. In the present study, we initially noticed that electrophoresis of HeLa cell extracts under non-reducing conditions (i.e., without DTT), followed by Western blotting, resulted in a rather fuzzy and more rapidly migrating form of SRPK1 than that observed under reducing conditions (i.e., with DTT) (Fig 2A). To exclude the possibility that this was a cell type-specific effect we repeated the electrophoretic and Western blotting analysis of SRPK1 using extracts from various cell lines. A similar shift of the SRPK1 band was detected in the absence of DTT, in all cell lines tested, suggesting the presence of disulfide bond(s) (Fig 2A). This was a rather unexpected finding since SRPK1 is mainly localized in the cytoplasm where the environment is reducing while disulfide bond formation generally occurs in the endoplasmic reticulum, due to its more oxidizing environment. Disulfide bonds can be formed in cytoplasmic proteins but usually under conditions of oxidative stress [24]. Even more unexpectedly, a clear, even though less conspicuous, shift was also observed following electrophoresis of bacterially produced, purified GST-SRPK1 in the absence of DTT (Fig 2B). Under physiological conditions, the *Escherichia coli* cytoplasm is maintained in a reduced state that strongly disfavors the formation of stable disulfide bonds in proteins [25, 26].

**Fig 3:**
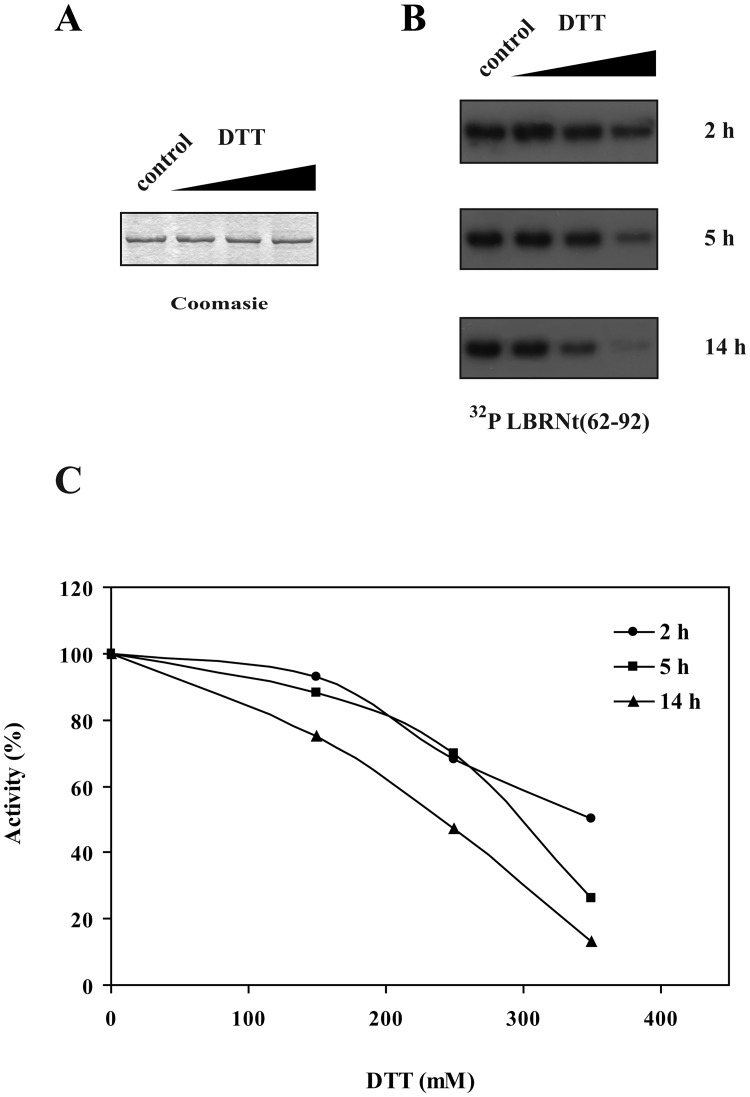
Effect of reducing agents on SRPK1 activity. (A) GST-SRPK1 was incubated with 0, 150, 250 and 350 mM DTT at room temperature for 14 h, then analyzed on 10% SDS-PAGE under reducing conditions and stained with Coomassie Blue. No degradation of GST-SRPK1 was observed following its overnight incubation with DTT. (B) GST-SRPK1 was incubated with 0, 150, 250 and 350 mM DTT at room temperature for 2, 5 and 14 h respectively and then used in kinase assays with GST-LBRNT(62–92) as substrate. Phosphorylated proteins were separated by 12% SDS-PAGE, stained with Coomassie Blue and autoradiographed. Only the relevant part of the autorad corresponding to phosphorylated GST-LBRNt(62–92) is shown. (C) Signals were quantified by excising the radioactive bands from the gel and scintillation counting. Measurements were done in triplicate. Error bars designate standard error.

We then wanted to investigate whether a possible conformational change of SRPK1, mediated by DTT, is related to an alteration of the kinase activity. In this respect, and as the disulfide bond(s) appear to be also present in the bacterially produced SRPK1, we incubated GST-SRPK1 with increasing concentrations of DTT and the remaining activity was assayed. When we incubated GST-SRPK1 with concentrations of DTT lower than 100 mM, for time periods up to 5 h, the activity of the kinase remained practically unaffected (Fig 3). However, incubation of GST-SRPK1, at room temperature, with concentrations of DTT higher than 100 mM and for longer time periods resulted in a progressive loss of activity (Fig 3). A similar loss of activity was also observed upon incubation of SRPK1 with β-mercaptoethanol (S1 Fig). The observation that an overnight incubation with 350 mM DTT was required for almost complete loss of activity indicated the existence of strong internal disulfide bonds that were not easily accessible to reducing agents.

### Evaluation of disulfide bond(s) location in the SRPK1 molecule

**Fig 4:**
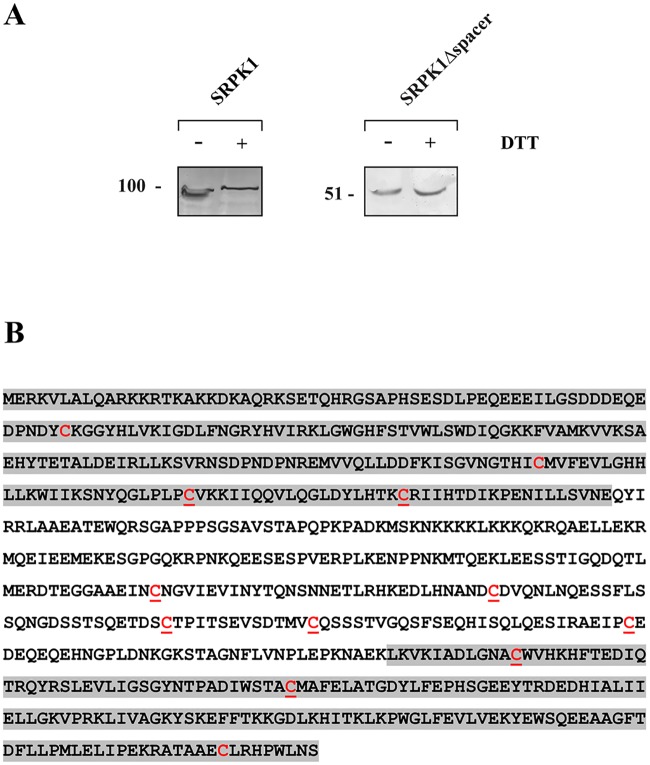
Evaluation of disulfide bond(s) location in the SRPK1 molecule. (A) Lysates from 293T cells overexpressing FLAG-SRPK1 or FLAG-SRPK1Δspacer were analyzed by SDS-PAGE, under non-reducing or reducing conditions, and Western blotting. SRPK1 was detected using the M5 anti-FLAG monoclonal antibody. (B) Amino acid sequence of SRPK1. The two catalytic domains are highlighted by grey shadows. All cysteines are marked in red; underlined cysteines were mutated to alanine or glycine.

As in all SRPK1 simplified models the spacer region is depicted as a loop, outside the kinase core (see Fig 1B), we hypothesized that such a loop structure might be stabilized by intramolecular disulfide bond(s). In this respect, we analyzed by SDS-PAGE, under both reducing and non-reducing conditions, and Western blotting a mutant SRPK1 lacking the spacer (SRPK1Δspacer). Indeed, the apparent electrophoretic mobility of this mutant was not affected by DTT (Fig 4A), thus suggesting the existence of disulfide bonds within the spacer region. SRPK1 contains 12 cysteine residues (Fig 4B), nine of which are located either within the spacer domain (Cys356, Cys386, Cys414, Cys427, Cys455) or in its proximal C-terminal (Cys188, Cys207) and N-terminal (Cys502, Cys539) ends of the two kinase catalytic domains, respectively. Based on the evidence provided by the SDS-PAGE and Western blotting analysis of SRPK1Δspacer and our assumption on the potential role of disulfide bond(s) in the proper folding of SRPK1, we decided to replace each of the nine cysteines and characterize their effect on SRPK1 activity and localization. As the spacer sequence is considered as a disordered segment, we substituted each of Cys356, Cys386, Cys414, Cys427 and Cys455 with glycine, instead of the more commonly used alanine, because glycine has a poor helix-forming propensity, whereas alanine has the highest [27]. We also took into consideration that glycine is a polar amino acid, while alanine has a rather hydrophobic side chain. Furthermore, since, according to the available crystal structure, Cys207 and Cys502 are also located in non-α-helical regions, we also substituted these two cysteines with glycine and only Cys188 and Cys539 that are found within α-helices were substituted with alanine (S2 Fig).

### Activity of SRPK1 cysteine mutants—Effect of reducing agents on their elecrophoretic mobility

**Fig 5:**
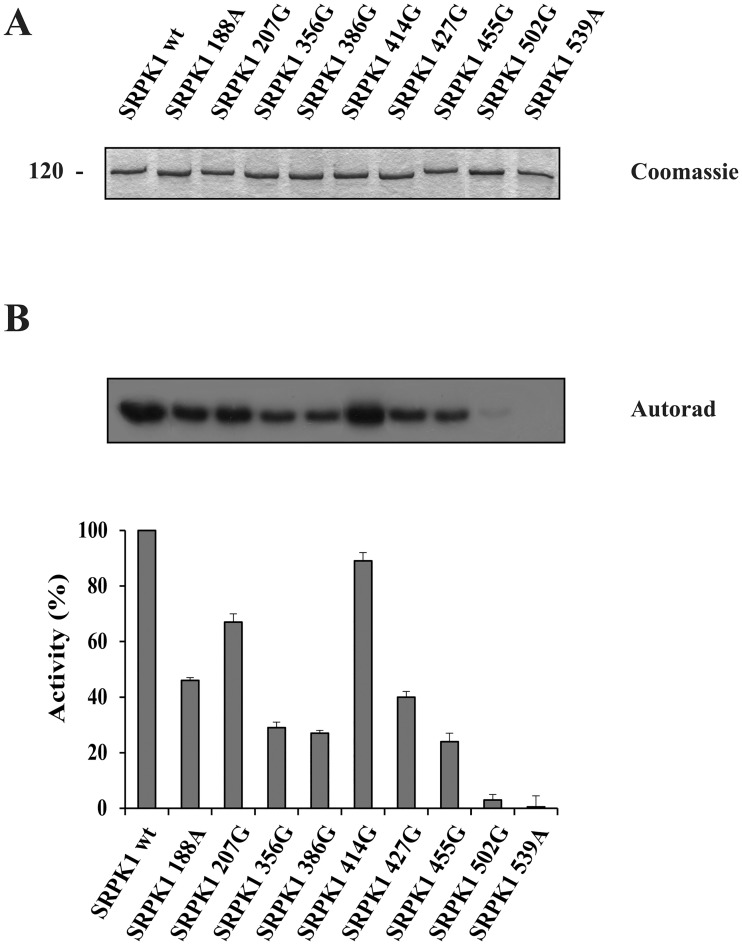
Influence of cysteine mutations on SRPK1 activity. Phosphorylation of GST-LBRNt(62–92) by 0.5 μg wild-type GST-SRPK1, GST-SRPK1 188A, GST-SRPK1 207G, GST-SRPK1 356G, GST-SRPK1 386G, GST-SRPK1 414G, GST-SRPK1 427G, GST-SRPK1 455G, GST-SRPK1 502G and GST-SRPK1 539A, respectively. (A) On top of the figure we show a Coomassie Blue staining of the recombinant GST-SRPK1 proteins (1.5 μg of each) used in the phosphorylation assays. (B) The samples were analyzed by SDS-PAGE on 12% gels, stained with Coomassie Blue and autoradiographed. The radioactive bands corresponding to GST-LBRNt(62–92) were excised and scintillation counted. Data represent the means ± SE of three independent experiments.

The wild-type and mutant SRPK1 were each expressed, initially in bacterial cells, as GST-fusion proteins, purified, and used for the *in vitro* phosphorylation of GST-LBRNt(62–92) (Fig 5A). To our surprise, since the spacer region is unnecessary for kinase activity, mutation of any cysteine residue within this domain, apart from Cys414, reduced SRPK1 activity to a significant extent (Fig 5B). Mutation of either of the two cysteines of the N-terminal catalytic domain (Cys188 or Cys207) was better tolerated as we observed a modest reduction of activity, whereas mutation of either of the two cysteines of the C-terminal catalytic domain (Cys502 or Cys539) practically abolished SRPK1 activity (Fig 5B).

To further substantiate these findings, wild-type SRPK1 and its derived mutants were overexpressed in 293T cells as FLAG-tagged proteins. At first, the level of expression of each protein was assessed in the cell lysate using a monoclonal antibody against the FLAG epitope. Interestingly, while all cysteine mutants of the spacer domain (Cys356, Cys386, Cys414, Cys427 and Cys 455) were each detected in the cell lysate at levels more or less comparable with that of wild-type SRPK1, the cysteine mutants of the kinase domain (Cys188, Cys207,C502 and C539) were each detected at lower levels (S3A Fig). It is also of note that some of the mutants displayed slightly different mobilities than wild-type SRPK1, despite the presence of DTT in the loading buffer. These divergent apparent protein sizes might be due to variations of the secondary and tertiary structure of the respective mutant proteins. *In vitro* kinase activity of wild-type and mutant FLAG-SRPK1 was evaluated using immunopurified proteins -from normalized cell lysates containing each equal amounts of SRPK1- and GST-LBRNt(62–92) as substrate. Similar results were more or less obtained as with the bacterial GST-proteins (S3B Fig).

**Fig 6:**
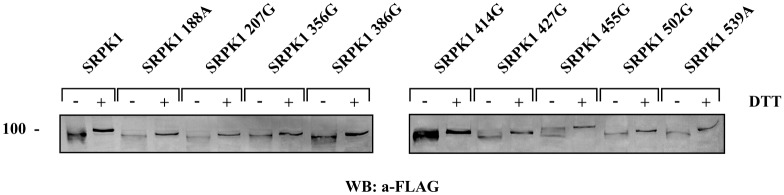
Effect of reducing agents on SRPK1 cysteine mutants electrophoretic mobility. Lysates from 293T cells transfected with wild-type SRPK1 and its mutant forms were analyzed under non-reducing conditions (i.e., without DTT) or reducing conditions (in the presence of 90 mM DTT) on 10% SDS-polyacrylamide gels. The proteins were then transferred to nitrocellulose and epitope-tagged wild-type or mutant SRPK1 was detected with the M5 anti-FLAG monoclonal antibody.

The requirement of all these cysteine residues for kinase activity strongly points to the formation of more than one disulfide bonds. To further support the idea of multiple disulfide linkages we tested the mobility of each of the point mutants in the presence and absence of DTT. As shown in Fig 6 the difference in the apparent electrophoretic mobility under non-reducing and reducing conditions of the respective mutant SRPK1 proteins still remained.

### Prediction of structural/functional consequences of cysteine mutations in the kinase domain

To investigate whether the observed effects of SRPK1 cysteine mutations are due to disulfide bond(s) disruption or are related to structural/functional alterations, we used the known crystal structure of the kinase domain of SRPK1 in complex with one of its substrates (PDB entry: 3BEG; [22]). Due to lack of structural data for the spacer region, as already mentioned, only the cysteines of the kinase domain (Cys188, C207, C502, and C539) were taken into consideration. As shown in S2 Fig, these four cysteines are located far away from each other. More precisely, the Cys-Cys distance is equal to 20.9 Å, for Cys188-Cys539; 18 Å, for Cys502-Cys539; 11.8 Å, for Cys207-Cys502 and 28.0 Å for Cys188-Cys207. These distances are much longer than the maximum distance (6.2 Å) required for disulfide bridging [28], implying that the formation of a disulfide bond between any of these cysteine pairs is unlikely. Thus, the observed reduction/loss of activity of the corresponding four mutants is most probably due to resulting structural/functional disturbances. Indeed, Cys502 is located at a position of the activation loop of SRPK1 which coincides with the start of a β-strand (β9) (S4 Fig, top left panel). Interestingly, the formation of this β-strand and of a resulting rigid β-sheet (β6-β9) is a highly conserved characteristic of the active state of kinases [29]. Replacement of Cys502 is therefore expected to prevent the formation of the β6-β9 sheet leading in turn to the deformation of the catalytic site, thus abolishing the activity of the kinase. This idea is in perfect agreement with the observed loss of activity of the SRPK1 502G mutant (Fig 5). On the contrary, substitution of Cys207, which is located in the vicinity of the afore-mentioned β6-β9 sheet (S4 Fig, top right panel), is expected to only marginally affect the rigidity of the β-sheet, in line with the observed moderate effect of the C207G mutation on the activity of the kinase (Fig 5).

The third cysteine of the Cys tetrad under consideration, C539, is part of a deep hydrophobic pocket of the docking groove of SRPK1 (S4 Fig, bottom), which is responsible for the accommodation of arginine residues at docking motif position 3 of SRPK1 substrates [30]. Substitution of this cysteine by an alanine residue is therefore predicted to distort the docking groove, through disruption of the packing of the hydrophobic residues forming the hydrophobic pocket (S4 Fig, bottom), thus interfering with substrate recognition. This prediction is in line with the observed loss of activity of the SRPK1 539A mutant (Fig 5).

Finally, the fourth cysteine, Cys188 is located at an α-helix far away from both the active site and the docking groove. Molecular dynamics simulations revealed that replacement of this cysteine by an alanine does not disturb the helical structure (S5 Fig). These observations, in conjunction with the C188A mutation data (Fig 5) and the proximity of Cys188 with the spacer region, support the idea that this particular cysteine may participate in disulfide bond formation with cysteine residues outside the SRPK1 kinase domain.

Taken together these observations strongly suggest that out of the four cysteines of the kinase domain only Cys188 may be involved in the formation of disulfide bonds in the active SRPK1 together with the cysteines of the spacer domain (apart from Cys414), whereas the effect of mutation of Cys502, Cys539 and probably Cys207 is structural.

### Nuclear translocation of SRPK1 and derived cysteine mutants

**Fig 7:**
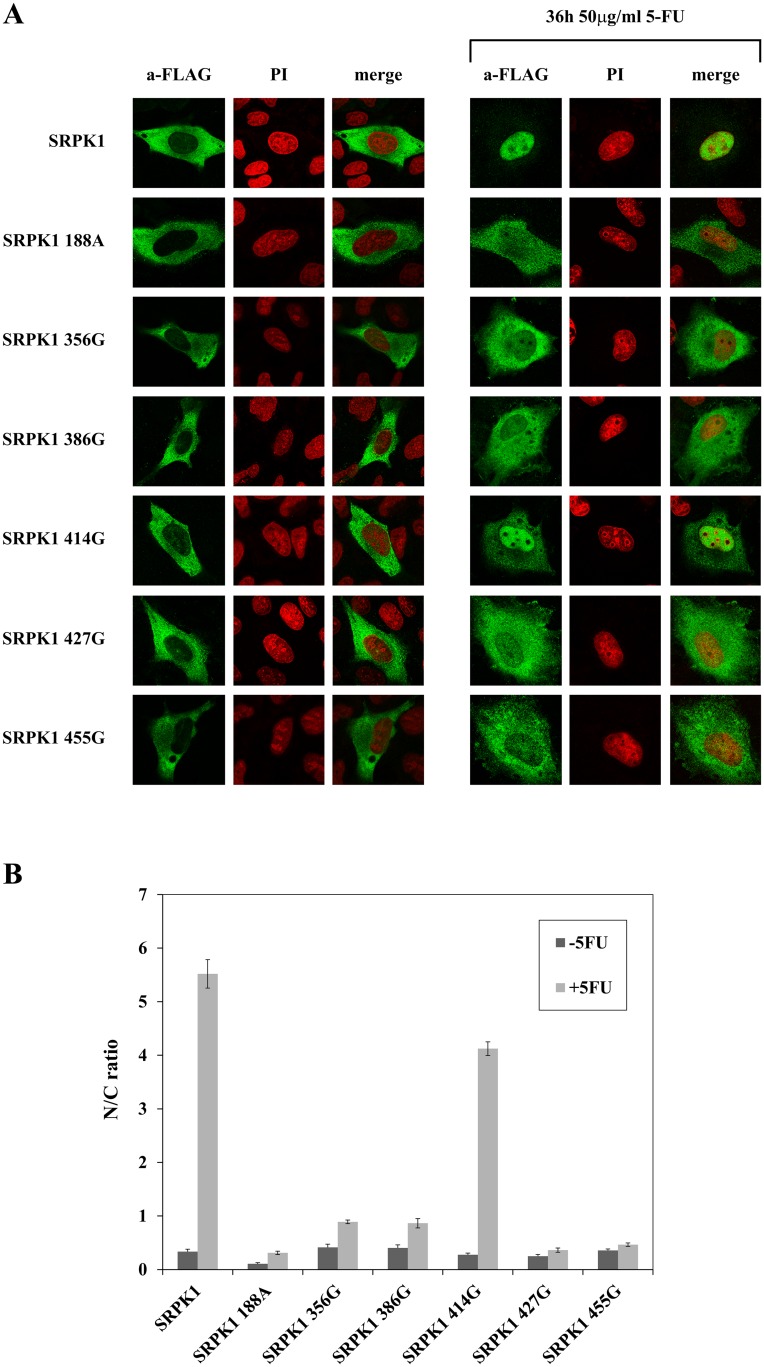
Nuclear translocation of SRPK1 and derived cysteine mutants in response to genotoxic stress. (A) Fluorescent pattern of wild-type FLAG-SRPK1 and mutant FLAG-SRPK1 188A, SRPK1 356G, SRPK1 386G, SRPK1 427G and SRPK1 455G in 5-FU-treated HeLa cells. SRPKs were detected using the M5 anti-FLAG monoclonal antibody, while nuclei were stained with propidium iodide (PI). Scale bar, 10 μm. (B) The ratio of average fluorescence intensity in the cell nucleus versus average intensity in the cell cytoplasm (N/C ratio) was quantified using ImageJ software. Each column represents the means ± SE of measurements from 20–30 cells.

SRPK1 is localized mainly in the cytoplasm of mammalian cells ([7]; see also Fig 7, left upper panel). It has been previously reported that in response to sorbitol-induced osmotic stress the kinase redistributes from the cytoplasm to the nucleus [15]. Yet, in our cells, nuclear translocation of exogenously expressed SRPK1 was less dramatically induced relative to this report (data not shown). As the nuclear amount of SRPK1 was also shown to increase upon treatment of H1299 cells with cisplatin [31], we performed a series of immunoflurescence experiments to identify genotoxic agents that might induce a strong nuclear accumulation of SRPK1 (Koutroumani and Giannakouros unpublished data). Among the agents tested, 5-fluorouracil (5-FU) was the most effective, resulting in almost complete nuclear translocation of SRPK1 (Fig 7, right upper panel).

We then proceeded to determine whether the lack or the reduced kinase activity of the cysteine mutants could affect their relocation from the cytoplasm to the nucleus, in response to 5-FU treatment. At first we checked the cysteine mutants of the spacer domain and SRPK1 188A that are most likely involved in disulfide linkage. As shown in Fig 7, while 5-FU triggered translocation of the active mutant SRPK1 414G to the nucleus (similarly to wild-type SRPK1), SRPK1 188A, SRPK1 356G, SRPK1 386G, SRPK1 427G and SRPK1 455G, which display reduced kinase activity, were less quantitatively shifted to the nucleus in 5-FU-treated cells. At this point it should be noted that while it was initially proposed that multiple elements of the spacer domain are required to regulate the localization of the kinase [7], our data clearly show that single mutations within the spacer are sufficient to modulate SRPK1 activity and hence its subcellular distribution.

Furthermore, and in agreement with our data, the phosphorylation-defective mutants SRPK1 502G and SRPK1 539A of the catalytic domain were fully restricted to the cytoplasm, whereas, 5-FU resulted in a limited translocation of SRPK1 207G to the nucleus (S6 Fig). These findings demonstrate that the efficiency by which the replacement of cysteine residues blocked the accumulation of SRPK1 in the nucleus of 5-FU-treated cells is closely related to the respective reduction of kinase activity and corroborate previous reports that the kinase activity is critical for the nuclear entrance of SRPK1 [7].

### Splicing efficiency of SRPK1 cysteine mutants

**Fig 8:**
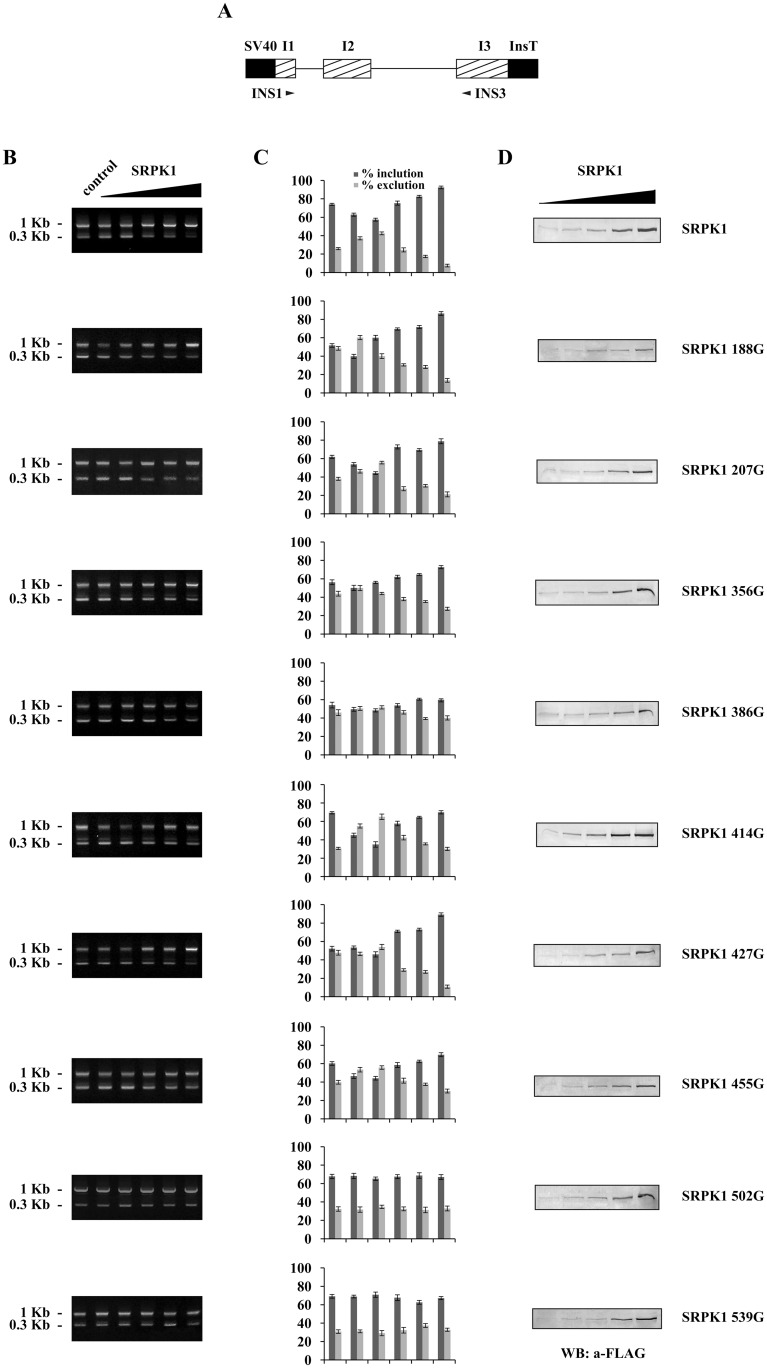
Impact of overexpressed wild-type and mutant SRPK1 on insulin reporter splicing. (A) Schematic representation of the insulin expression construct containing 2 introns and the respective flanking exons I1, I2 and I3. SV40 promoter/enhancer regions and insulin transcription terminators are shown in black. Primers used for PCR are indicated. (B) HeLa cells were transfected with increasing concentrations (0, 0.25, 0.3, 0.35, 0.5 and 0.7 μg) of plasmids expressing wild-type and mutant SRPK1 along with 1.65 μg of the reporter gene. Cells were treated with 50 μg/ml 5-FU for 24 h, prior harvesting. RNA was isolated 48 h following transfection, and RT-PCR was carried out. The spliced and unspliced products have a size of 0.3 and 1 kb respectively. (C) The ratio of the upper and lower bands was quantified using ImageJ software. Data represent the means ± SE of two independent experiments. (D) Western blots showing the levels of wild-type and mutated SRPK1.

One of the main functions of SRPK1 in the nucleus is to modulate splicing [1, 2]. It has been reported that nuclear translocation of SRPK1 mediated by stress signals is closely related to alterations in the splicing machinery [7, 15]. To determine whether mutation of the cysteine residues, besides cytoplasmic sequestration of SRPK1, also triggered additional events related to splicing efficiency, we co-transfected a reporter gene with increasing amounts of SRPK1 and its derived mutants. As reporter gene we used a rat insulin minigene that contains exons 1, 2 and 3 and the respective introns fused with the appropriate SV40 promoter/enhancer regions and insulin transcription terminators respectively (Fig 8A; see also [21]). In the absence of the stress signal (5-FU) and exogenously added SRPK1, a hardly detectable amount of the spliced insulin mRNA was observed (data not shown), consistent with the cytoplasmic accumulation of SRPK1 (Fig 7). When HeLa cells were treated with 5-FU and co-transfected with 0.25–0.3 μg of plasmid DNA encoding FLAG-SRPK1, we noted an induction of the spliced reporter mRNA as compared to 5-FU-treated cells but without overexpression of FLAG-SRPK1 (Fig 8B). Interestingly, transfection of 5-FU-treated HeLa cells with quantities of pFLAG-SRPK1 larger than 0.35 μg, resulted in the accumulation of the unspliced insulin pre-mRNA (Fig 8B), which might be due to splicing inhibition by an excess of nuclear SRPK1 leading presumably to hyperphosphorylation of SR proteins [7, 14, 15].

We then used the same insulin splicing reporter to determine its response to SRPK1 cysteine mutants. Consistent with previous observations [15] the kinase activity was required for the observed splicing effects, as the inactive SRPK1 502G and SRPK1 539A catalytic domain mutants practically failed to induce any changes in the splicing of the reporter RNA (Fig 8B and 8C). On the other hand, SRPK1 414G, which was as active as wild-type SRPK1, had a rather similar effect. The spacer domain cysteine mutants as well as SRPK1 188A and SRPK1 207G exhibited a modest effect, more or less analogous to their activity and nuclear translocation following 5-FU treatment (Fig 8B and 8C; see also Figs 5 and 7).

## Discussion

The available structural data on SRPK1 were based on the crystallization of an active fragment of SRPK1 (SRPK1ΔNS1) that lacks almost the entire spacer domain ([8]; see also Fig 1). In this truncated SRPK1 the adjacency of the two catalytic domains and the entailed functional interactions between the two lobes which are indispensable for the enzyme to adopt an active conformation [2] were “artificially” obtained by omitting the spacer region. Yet, in the entire SRPK1 molecule, the proximity of two catalytic domains can only be achieved if the spacer region (which is almost as large as the two catalytic domains together) twists into a loop-like structure. It is well-known that the formation of disulfide bridges is an important aspect of the stabilization of protein tertiary structure, allowing parts of the protein chain to be held together [32, 33]. In the present study we provide evidence that disulfide bonding of cysteine residues most probably stabilizes such a loop-like structure of the spacer region and is critical for SRPK1 structure and function.

According to our data, reducing agents affected the mobility of SRPK1 in SDS-gels and more importantly had a profound inhibitory effect on kinase activity. Furthermore, single cysteine substitutions within the spacer domain, except SRPK1 414G, showed significantly reduced kinase activity, thus making clear that the spacer domain is absolutely required for SRPK1 activity. Replacement of the four proximal cysteines located either at the first (Cys188, Cys207) or the second (Cys502 and Cys539) catalytic domain of SRPK1 also resulted in a loss/reduction of activity. According to predictions based on the available crystal structure of SRPK1, Cys502 and Cys539 seem to be critical for the kinase to acquire an active conformation and therefore their substitution resulted in a total loss of activity, Cys207 partially affects the configuration of the catalytic site, and only Cys188 which is located far away from both the active site and the docking groove may participate in disulfide bonding with the cysteines of the spacer domain. The requirement of several cysteine residues for kinase activity, combined with the observation that the difference in the apparent electrophoretic mobility under non-reducing and reducing conditions of the respective mutant SRPK1 proteins still remained (Fig 6), strongly point to the formation of more than one disulfide bonds. The strong reducing conditions required for inactivation of SRPK1 (see Fig 3) further strengthen this suggestion.

At present, it remains obscure how many and which cysteines participate in disulfide bond formation. Various servers for predicting the disulfide bonding state of cysteines gave different and more or less conflicting results. According to DISULFIND (http://disulfind.dsi.unifi.it/process.php) [34] and EDBCP: **E**nsemble-based **D**isulfide **B**onding **C**onnectivity **P**attern prediction server (http://biomedical.ctust.edu.tw/edbcp/) [35], SRPK1 does not seem to contain any intra-chain disulfide bridges. According to CYSPRED (http://gpcr.biocomp.unibo.it/cgi/predictors/cyspred/pred_cyspredcgi.cgi) [36], Cys386, Cys414 and Cys427 might be involved in disulfide bonding, with 414–427 being the most likely disulfide bond formed. However, the fact that Cys414 is redundant for SRPK1 activity invalidates this prediction. Finally, the DiANNA 1.1 web server (http://clavius.bc.edu/~clotelab/DiANNA/) [37] predicts the formation of multiple disulfide bonds, involving Cys188, Cys356, Cys386, Cys427 and Cys455. Interestingly, according to this server Cys207, Cys502 and Cys539 can also be found in a bonding state, a prediction which appears to be strongly unlikely according to our data and the crystal structure of SRPK1.

Related to the importance of all these cysteines for kinase activity and the likely involvement of the spacer domain cysteines and Cys188 in disulfide bonding we performed a search to see how conserved these residues are across multiple species by aligning the corresponding protein sequences. As previously reported the catalytic domains are highly conserved throughout evolution, whereas the spacer regions are very different both in their primary sequence and length [1]. In agreement with these observations Cys502, Cys539 and Cys207 are fully conserved among diverse species (S1 Table). Cys188 which according to the crystal structure is not critical for kinase activity and may participate in disulfide bridges is the less conserved catalytic cysteine (S1 Table). Interestingly, drosophila and worm that lack Cys188 have instead a nearby cysteine residue at positions 170 and 193, respectively (numbering according to human SRPK1). While the five cysteines of the spacer domain are fully conserved among mammals (with the exception of Cys455 which, out of forty five mammalian sequences tested, is not present only in mouse and rat SRPK1) their percentage of conservation varies among the SRPK1 sequences that we randomly examined from different groups of organisms. Cys356 and Cys386 are more conserved, while Cys414, Cys427 and Cys455 are less conserved (S1 Table). Due to the high variability of the spacer sequences various species lack some of the spacer domain cysteines found in mammalian SRPK1 but instead they have other cysteine residues that may substitute their function. For example, birds lack Cys414 and Cys455 but they have cysteine residues at positions 350 and 396, reptiles lack Cys455 but they have a cysteine residue at position 350, fish lack Cys386, Cys414, Cys427 and Cys455 but they have cysteine residues at positions 285, 314 and 401 (numbering according to human SRPK1). However, it is rather the number of cysteines in relation to the length of the spacer region that is critical than their percentage of conservation. Indicatively, zebrafish srpk1, saccharomyces cerevisiae Sky1, worm Spk-1, drosophila Srpk79D and saccharomyces pombe Dsk1 that have a 28, 28, 33, 60 and 149 amino acids, respectively, shorter spacer region than human SRPK1 contain four, four, three, two and one cysteine residues, respectively, instead of five (human SRPK1). It seems therefore that the number of cysteine residues within the spacer region is proportional to its length, implying that shorter spacer regions may require lesser disulfide bonds.

Besides the characterization of possible disulfide bonds, other interesting issues remain to be addressed. First, how these bonds are formed. Cytoplasmic proteins, in general, do not contain disulfide bonds because the sulfhydryl groups in the vast majority of protein cysteine residues (Cys-SH) have a p*Ka* > 8.0 and, in the reducing environment of the cytoplasm, remain protonated [38]. The formation of disulfide bridges is only likely when certain proteins possess cysteine residues that exist as thiolate anions at neutral pH due to a lowering of their p*Ka* values by charge interactions with neighboring amino acid residues [38, 39]. It remains to be seen by structural studies whether the cysteines of the spacer domain become adjacent to such a charged microenvironment. In this respect it is tempting to speculate that molecular chaperones may not only act to sequester SRPK1 in the cytoplasm but they may also provide the proper environment to facilitate the formation of disulfide linkages. Furthermore, as it is clear nowadays that disulfide bonds have been added to proteins not only to help hold them together, but also as a way of controlling how they work [40, 41], a second important issue is whether the SRPK1 molecule contains a fixed set of disulfide bonds which are inert; that is, once formed, they remain unchanged for the lifetime of the kinase. An intriguing hypothesis would be that a flexible bonding network with the ability to remodel may function as an activity “rheostat” for SRPK1 by modulating the bending of the spacer region and subsequently the proximity of the two catalytic domains. Contingent catalysts or facilitators that mediate the cleavage of specific bonds remain to be identified.

In conclusion, we provide evidence for a new potential mode of regulation of SRPK1 through the formation of disulfide bonds. Correctly characterizing the disulfide bond topology in SRPK1 will be of crucial importance for understanding its structural and functional characteristics.

## Supporting information

S1 Fig Effect of mercaptoethanol on SRPK1 activity. GST-SRPK1 was incubated with 350 mM DTT or 700 and 1250 mM mercaptoethanol (ME) at room temperature for 14 h and then used in kinase assays with GST-LBRNT(62–92) as substrate. Phosphorylated proteins were separated by 12% SDS-PAGE, stained with Coomassie Blue and autoradiographed. Only the relevant part of the autorad corresponding to phosphorylated GST-LBRNT(62–92) is shown.
(TIF)

S2 Fig Ribbon representation of the known SRPK1 crystal structure (PDB entry: 3BEG) lacking the spacer domain and the N-terminal region 1–68. The SRPK1 ribbon model is colored according to its secondary structure elements (red, yellow, green for α-helices, β-strands and loops, respectively). Cys207 and Cys502 are located in non-α-helical regions, while Cys188 and Cys539 are found within α-helices. The displayed distances between Cys188, Cys207, Cys502 and Cys539 (see text) illustrate the incompatibility of the active SRPK1 conformation with the existence of disulphide bonds between cysteine pairs of this domain.
(TIF)

S3 Fig Influence of cysteine mutations on the activity of FLAG-SRPK1. (A) Expression of FLAG-SRPK1 and its derived cysteine mutants. Lysates from 293T cells transfected with wild-type SRPK1 and its mutant forms and containing equal amount of total protein were analyzed on 10% SDS-polyacrylamide gels. The proteins were then transferred to nitrocellulose and epitope-tagged wild-type or mutant SRPK1 was detected with the M5 anti-FLAG monoclonal antibody. (B) Kinase activity of FLAG-SRPK1 and its derived cysteine mutants. 293T cells were transfected with wild-type FLAG-SRPK1 and its mutant forms. Anti-FLAG immunoprecipitates from normalized cell lysates, containing each equal amounts of SRPK1, were subjected to an *in vitro* kinase assay with GST-LBRNt(62–92) as substrate in the presence of [γ-^32^P] ATP. The samples were analyzed by SDS-PAGE on 12% gels, stained with Coomassie Blue and autoradiographed. The radioactive bands corresponding to GST-LBRNt(62–92) were excised and scintillation counted. Data represent the means ± SE of three independent experiments.
(TIF)

S4 Fig Details of the region of Cys502, Cys207 (top panel) and Cys539 (bottom panel) in the known crystal structure of the SRPK1 kinase domain in complex with a substrate peptide. (Top) SRPK1 is depicted as ribbon model and colored according to its secondary structure elements as in S2 Fig. Only important residues and residues discussed in the text are shown (in sticks), for clarity. Asterisks denote catalytic aspartates. (Bottom) The docking domain of SRPK1 is depicted as ribbon model and as a surface colored by the electrostatic potential, whereas the substrate peptide is colored in green (left panel). Cys539 and the arginine at docking motif position 3 [30] of the substrate peptide are depicted as ball-and-sticks. Other hydrophobic residues of SRPK1 participating in the formation of the deep hydrophobic pocket of its docking groove, are shown in sticks (right panel). This figure was produced using coordinates from the PDB entry: 3BEG [22].
(TIF)

S5 Fig Substitution of Cys188 with alanine does not distort the respective α-helix. Helix 3 (aa 185–207) and its direct environment of mutant SRPK1 C188A after 20 ns of MD simulation (colored) compared to that of wild type SRPK1 (grey).
(TIF)

S6 Fig Nuclear translocation of SRPK1 and derived catalytic domain cysteine mutants in response to genotoxic stress. (A) Fluorescent pattern of wild-type FLAG-SRPK1 and mutant FLAG-SRPK1 502G, FLAG-SRPK1 539A, and FLAG-SRPK1 207G in 5-FU-treated HeLa cells. SRPKs were detected using the M5 anti-FLAG monoclonal antibody, while nuclei were stained with propidium iodide (PI). Scale bar, 10 μm. The induced by 5-FU redistribution of SRPK1 from the cytoplasm to the nucleus is closely related to the kinase activity. (B) (B) The ratio of average fluorescence intensity in the cell nucleus versus average intensity in the cell cytoplasm (N/C ratio) was quantified using ImageJ software. Data represent the means ± SE of measurements from 25–30 cells.
(TIF)

S1 Table Conservation of SRPK1 cysteines across various classes of species. The amino acid sequences of SRPK1 from mammals (45 species), birds (8 species), reptiles (8 species), amphibia (xenopus), fish (5 species), insects (fruit fly), worm, yeast (saccharomyces cerevisiae and saccharomyces pombe) were aligned by the ClustalO. Conserved cysteines across various classes are denoted by a thick dot, while non conserved cysteines are denoted by a blank space.
(DOC)
