# Supplementary figures and images for: Evidence for disulfide bonds in SR Protein Kinase 1 (SRPK1) that are required for activity and nuclear localization
Source: PLoS One. 2017 Feb 6;12(2):e0171328. doi: 10.1371/journal.pone.0171328 (PMC5293202; doi:10.1371/journal.pone.0171328)

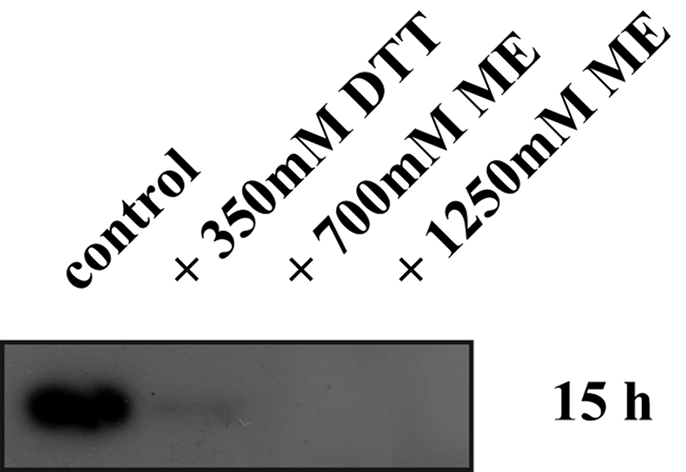

Supplement: S1 Fig — GST-SRPK1 was incubated with 350 mM DTT or 700 and 1250 mM mercaptoethanol (ME) at room temperature for 14 h and then used in kinase assays with GST-LBRNT(62–92) as substrate. Phosphorylated proteins were separated by 12% SDS-PAGE, stained with Coomassie Blue and autoradiographed. Only the relevant part of the autorad corresponding to phosphorylated GST-LBRNT(62–92) is shown. (TIF) [file pone.0171328.s001.tif]

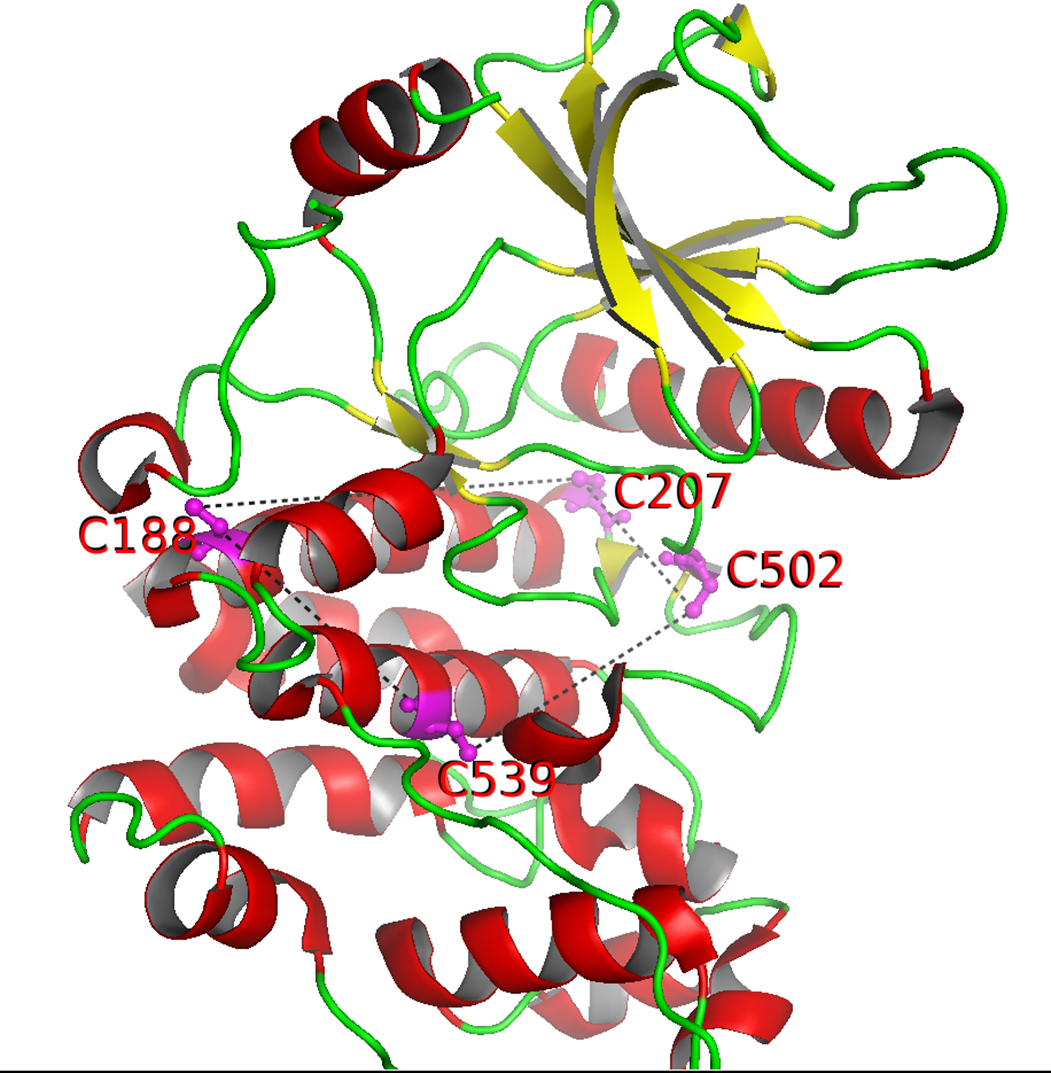

Supplement: S2 Fig — The SRPK1 ribbon model is colored according to its secondary structure elements (red, yellow, green for α-helices, β-strands and loops, respectively). Cys207 and Cys502 are located in non-α-helical regions, while Cys188 and Cys539 are found within α-helices. The displayed distances between Cys188, Cys207, Cys502 and Cys539 (see text) illustrate the incompatibility of the active SRPK1 conformation with the existence of disulphide bonds between cysteine pairs of this domain. (TIF) [file pone.0171328.s002.tif]

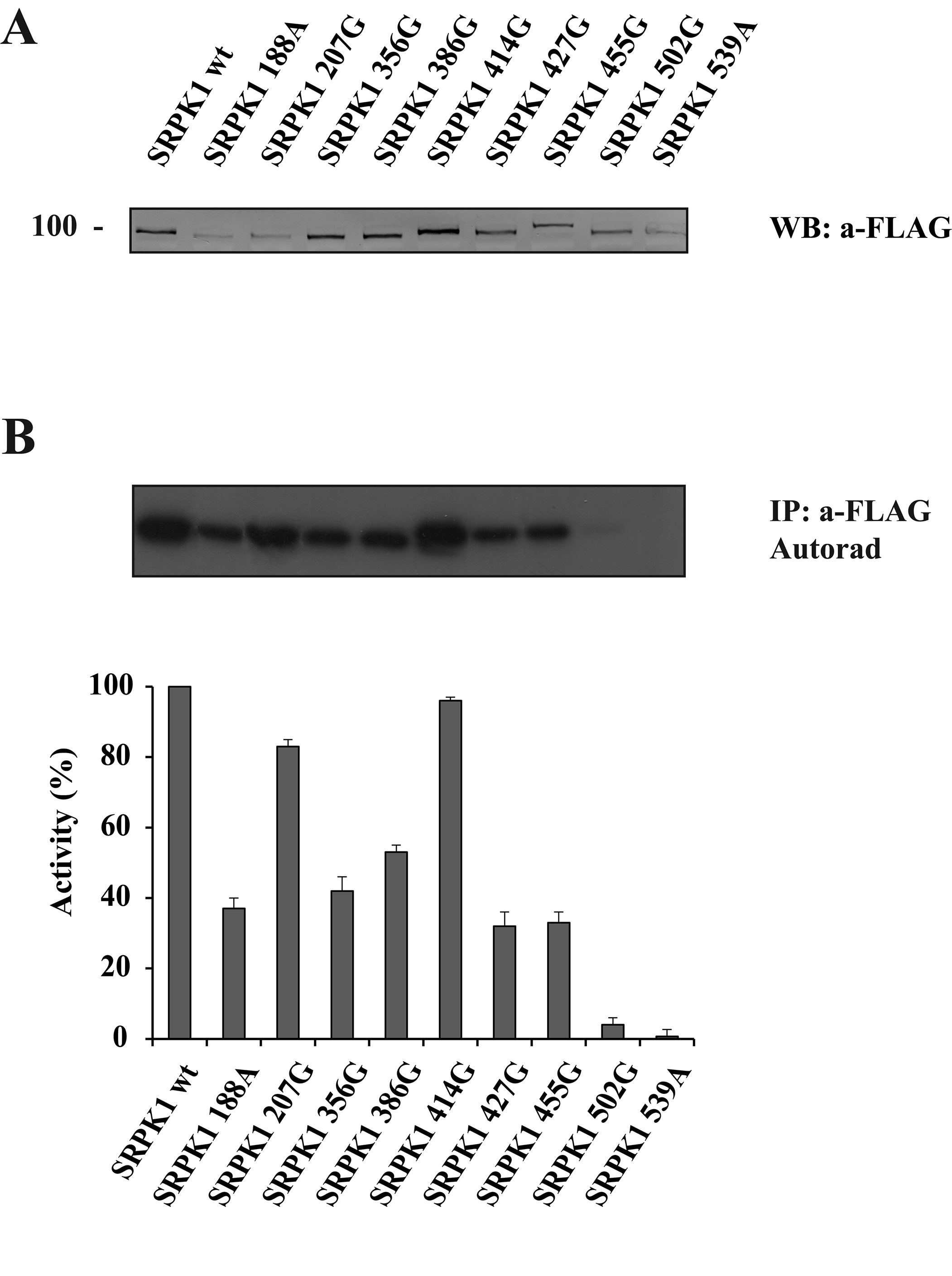

Supplement: S3 Fig — (A) Expression of FLAG-SRPK1 and its derived cysteine mutants. Lysates from 293T cells transfected with wild-type SRPK1 and its mutant forms and containing equal amount of total protein were analyzed on 10% SDS-polyacrylamide gels. The proteins were then transferred to nitrocellulose and epitope-tagged wild-type or mutant SRPK1 was detected with the M5 anti-FLAG monoclonal antibody. (B) Kinase activity of FLAG-SRPK1 and its derived cysteine mutants. 293T cells were transfected with wild-type FLAG-SRPK1 and its mutant forms. Anti-FLAG immunoprecipitates from normalized cell lysates, containing each equal amounts of SRPK1, were subjected to an in vitro kinase assay with GST-LBRNt(62–92) as substrate in the presence of [γ-32P] ATP. The samples were analyzed by SDS-PAGE on 12% gels, stained with Coomassie Blue and autoradiographed. The radioactive bands corresponding to GST-LBRNt(62–92) were excised and scintillation counted. Data represent the means ± SE of three independent experiments. (TIF) [file pone.0171328.s003.tif]

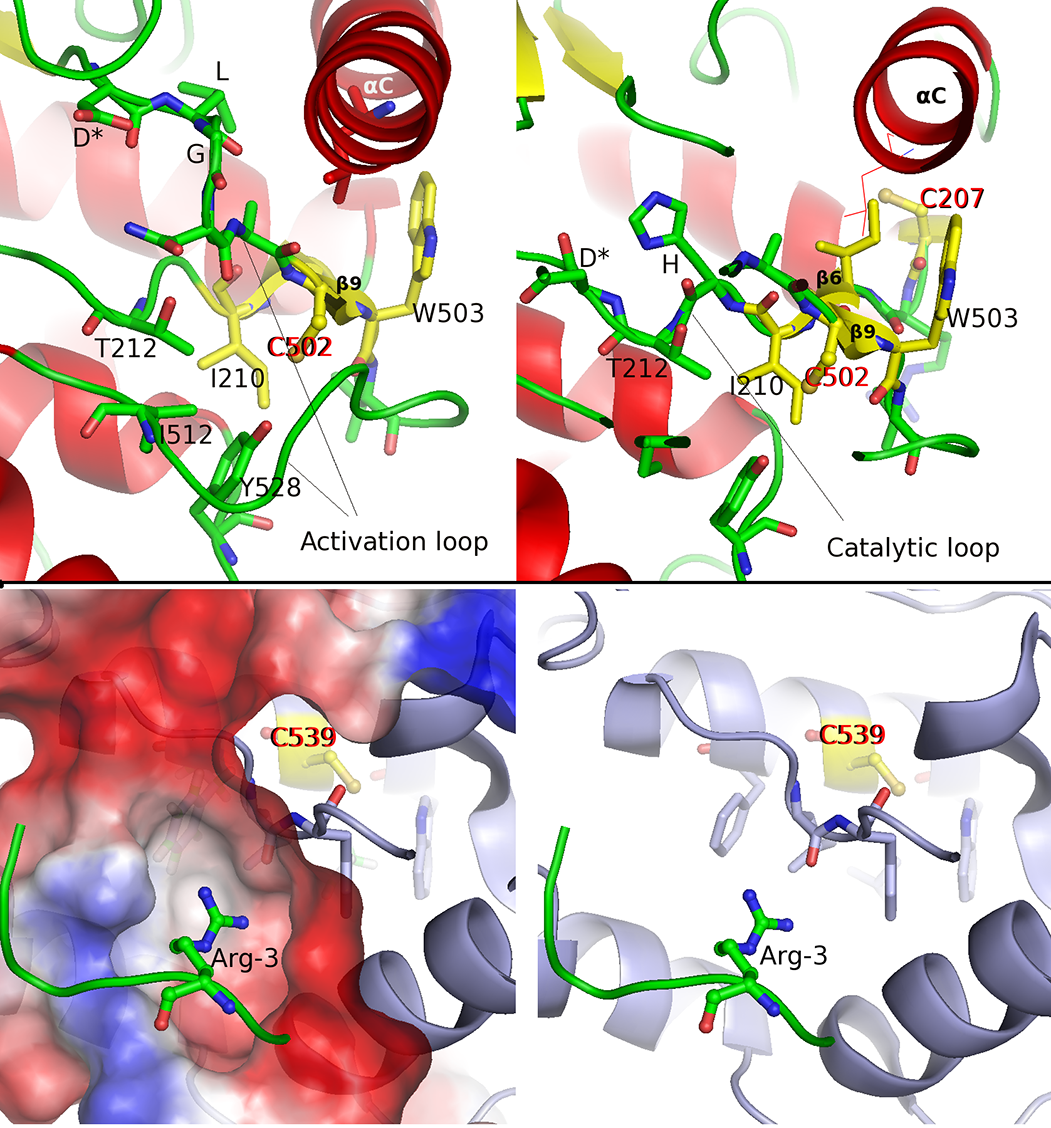

Supplement: S4 Fig — (Top) SRPK1 is depicted as ribbon model and colored according to its secondary structure elements as in S2 Fig. Only important residues and residues discussed in the text are shown (in sticks), for clarity. Asterisks denote catalytic aspartates. (Bottom) The docking domain of SRPK1 is depicted as ribbon model and as a surface colored by the electrostatic potential, whereas the substrate peptide is colored in green (left panel). Cys539 and the arginine at docking motif position 3 [30] of the substrate peptide are depicted as ball-and-sticks. Other hydrophobic residues of SRPK1 participating in the formation of the deep hydrophobic pocket of its docking groove, are shown in sticks (right panel). This figure was produced using coordinates from the PDB entry: 3BEG [22]. (TIF) [file pone.0171328.s004.tif]

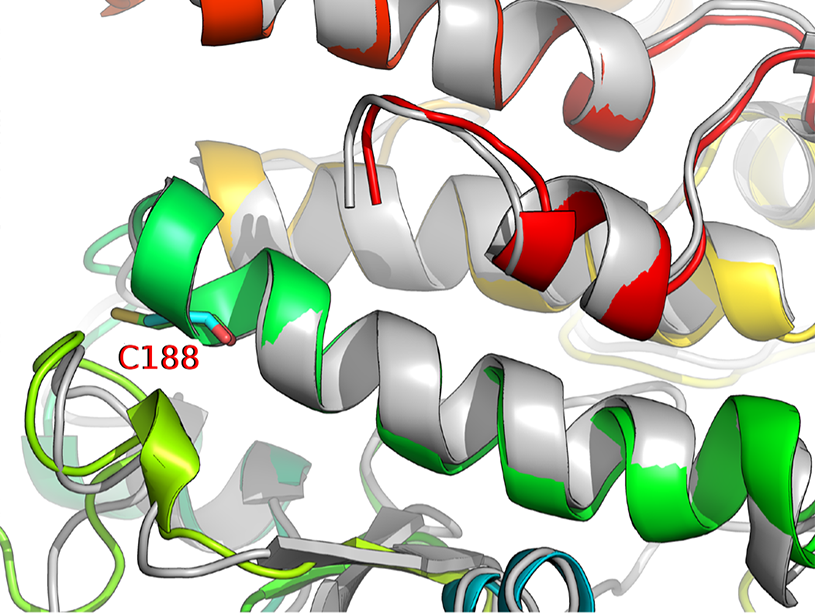

Supplement: S5 Fig — Helix 3 (aa 185–207) and its direct environment of mutant SRPK1 C188A after 20 ns of MD simulation (colored) compared to that of wild type SRPK1 (grey). (TIF) [file pone.0171328.s005.tif]

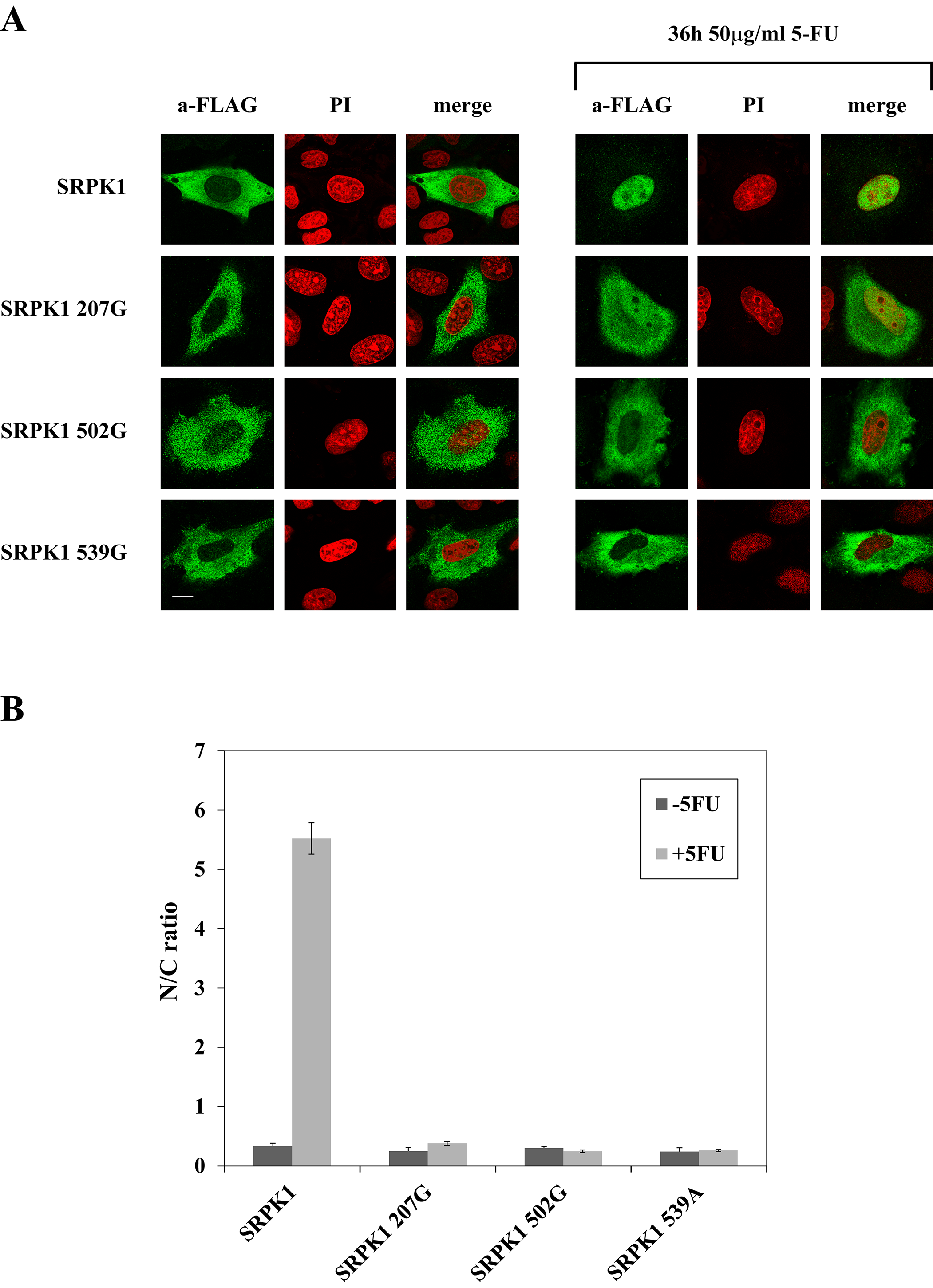

Supplement: S6 Fig — (A) Fluorescent pattern of wild-type FLAG-SRPK1 and mutant FLAG-SRPK1 502G, FLAG-SRPK1 539A, and FLAG-SRPK1 207G in 5-FU-treated HeLa cells. SRPKs were detected using the M5 anti-FLAG monoclonal antibody, while nuclei were stained with propidium iodide (PI). Scale bar, 10 μm. The induced by 5-FU redistribution of SRPK1 from the cytoplasm to the nucleus is closely related to the kinase activity. (B) (B) The ratio of average fluorescence intensity in the cell nucleus versus average intensity in the cell cytoplasm (N/C ratio) was quantified using ImageJ software. Data represent the means ± SE of measurements from 25–30 cells. (TIF) [file pone.0171328.s006.tif]
