# Supplementary material for: Evidence for disulfide bonds in SR Protein Kinase 1 (SRPK1) that are required for activity and nuclear localization
Source: PLoS One. 2017 Feb 6;12(2):e0171328. doi: 10.1371/journal.pone.0171328 (PMC5293202; doi:10.1371/journal.pone.0171328)
Supplement: S1 Table — The amino acid sequences of SRPK1 from mammals (45 species), birds (8 species), reptiles (8 species), amphibia (xenopus), fish (5 species), insects (fruit fly), worm, yeast (saccharomyces cerevisiae and saccharomyces pombe) were aligned by the ClustalO. Conserved cysteines across various classes are denoted by a thick dot, while non conserved cysteines are denoted by a blank space. (DOC) [file pone.0171328.s007.doc]

| **Cysteines** | **188** | **207** | **356** | **386** | **414** | **427** | **455** | **502** | **539** |
| --- | --- | --- | --- | --- | --- | --- | --- | --- | --- |
| **Mammals** | • | • | • | • | • | • | • | • | • |
| **Birds** | • | • | • | • |  | • |  | • | • |
| **Reptiles** | • | • | • | • | • | • |  | • | • |
| **Amphibia** | • | • | • | • | • |  | •  | • | • |
| **Fish** | • | • | • |   |  |  |  | • | • |
| **Drosophila** | 170 | • | • | • |  |  |  | • | • |
| **Worm** | 193 | • | • |  |  |  |  | • | • |
| **Yeast** |  | • |  |  |  |  |  | • | • |
